# A mental health intervention program for the oocyte donors: protocol for a mixed methods study

**DOI:** 10.1186/s12978-020-0864-9

**Published:** 2020-01-20

**Authors:** Elham Adib Moghaddam, Ashraf Kazemi, Gholamreza Kheirabadi, Seyyed Mehdi Ahmadi

**Affiliations:** 1 Student Research Committee, School of Nursing and Midwifery, Isfahan University of Medical Sciences, Isfahan, Iran; 2 Nursing and Midwifery Care Research Center, School of Nursing and Midwifery, Isfahan University of Medical Sciences, Isfahan, Iran; 3 Reproductive Health department, Isfahan University of Medical Sciences, Hezarjerib AV, Isfahan, Iran; 4 Behavioral Sciences Research Center, Isfahan University of Medical Sciences, Isfahan, Iran; 5 Isfahan Fertility and Infertility Center, Isfahan, Iran

**Keywords:** Mental health, Oocyte donation, Assisted reproductive technique

## Abstract

**Background:**

Oocyte donation is one of the assisted reproductive techniques that can undermine the mental health of the women donor. As such, the aim of this study is to design a mental health promotion program for oocyte donors.

**Methods:**

This is an exploratory mixed methods study (qualitative-quantitative) that consists of three phases. In the first phase, a qualitative study will be conducted to identify the needs and strategies of the mental health promotion program for the women donors. In this phase, the participants will be selected using purposeful sampling method and the data will be collected through semi-structured interviews. In the second phase, the initial draft of the program is designed and validated in the panel of experts and using the classic Delphi technique and, then, the program is finalized. In the third phase, the designed program will be implemented as a quasi-experimental study in two groups of program recipients (intervention) and control, and the effectiveness of the intervention program will be evaluated.

**Discussion:**

In order to design a documentation-based mental health promotion program for the oocyte donors, their experience during the process of oocyte donation should be evaluated. Doing so, the program will be developed based on the specific circumstances of the target population and their social and cultural context and, hence, will have the highest impact.

## Plain English summary

Oocyte donation is both psychologically and morally so challenging for the donors. Given the mental complications caused by oocyte donation and considering ethical principles in assisted reproductive treatments, an intervention program seems necessary to maintain, provide and promote the mental health of the donors. The present research will provide some comprehensive information regarding the needs and strategies for improving the psychological health in the oocyte donors. Using an exploratory mixed method, this study will be conducted in three phases. In the first phase, a qualitative study will be conducted to extract the needs and strategies of the mental health program for the oocyte donors. The participants in this phase will be selected using purposive sampling method and the data will be collected using semi-structured interviews and taking notes in the field. The data will be analyzed using the conventional content analysis method. In the second phase, using the information obtained from the previous phase and review of the literature, the initial draft of the mental health program for the oocyte donors will be prepared. Then, using classic Delphi technique, the program will be validated and finalized in the panel of experts based on the priorities. In the third phase, the designed program will be implemented using a quantitative approach, and the effectiveness of the program will be evaluated comparing the mental health status of the two groups of donors before and after the implementation of the program. As such, this program designed based on a qualitative study and review of the literature and the newest information, can improve the mental health of the oocyte donors.

## Background

The use of donated oocyte in assisted reproductive technology (ART) is an accepted method in the treatment of the couples who cannot be pregnant through autologous oocyte [1]. The use of this technique is increasingly growing so that 10% of all ART cycles in 2017 in the United States have been performed using oocyte donation [2].

In this method, the whole processes of the ovarian stimulation, ovarian response monitoring and the oocyte retrieval are performed on the oocyte donors and, after in vitro fertilization or intracytoplasmic sperm injection procedure, the resulted embryo or embryos are transferred to the uterus of the recipient woman [3]. In this process, the recipient of the donated oocyte benefits from having a child, but the donor, after suffering from possible problems and complications of the ovarian stimulation and oocyte retrieval, is only provided with humanitarian feelings and financial compensation [4, 5].

This experience had not been satisfying for many donors [6]. In addition to tolerating the perils of the ART process, the donors are also supposed to bear the consequent social stigma for entering this procedure and face socio-cultural taboos in traditional societies [6–8]. Because, reproduction in many cultures is a private issue defined in the form of marital relations, and the participation of a third-party is not accepted in it [9].

Materialistic use of the body for gaining financial benefits is another related issue [10]. Another social challenge is the likelihood of contact between the donor and the resulted child in the future [11]. Taken together, these issues can turn a process which can be associated with a good sense of altruistic help into an unpleasant experience endangering the mental health of the oocyte donor.

Previous studies also show a decline in the mental health of the donors during this process [8, 12, 13]. As such, these women need to be considered as one of the target groups of the mental health promotion programs in the health systems of different countries. However, in many ART service centers, attentions are limited to screening for the psychological disorders of the oocyte donors before entering the donation process [14].

Among the Muslim countries, Iran is the only country in which using a third-party and oocyte donation is allowed in the process of reproduction [15], and many volunteers of using donated oocyte in other neighboring countries such as Iraq and Afghanistan refer to ART service centers in Iran to receive treatment. Nonetheless, there is no appropriate program to maintain, supply and promote the psychological condition of the oocyte donors. Therefore, the design of a program for maintaining the psychological health of the oocyte donors seems to be necessary. Accordingly, an exploratory mixed method study will be designed and performed in order to achieve an intervention program appropriate for the socio-cultural conditions of the country.

## Methods

This study approved by the Ethics Committee of Isfahan University of Medical Sciences in Isfahan, Iran (IR.MUI.RESEARCH.REC.1398.492), and will be conducted in the centers for providing ART services in Isfahan. Informed consent will be obtained from the participants at all phases of the research. The design of the program will be based on the Talbot and Verrinder model and will be implemented in three phases.

The first step of this programing pattern includes the analysis of the needs and determination of a specific subject for programing. The draft of the program will be designed in the second step; then, the finalization of the program, its implementation, monitoring of the implementation and evaluation of the effectiveness of the program will be performed in the third to sixth steps respectively [16]. In the first phase of the study that is in line with the first step of the programing pattern, using content analysis method, a qualitative study will be conducted to identify the needs and strategies of the program.

In the second phase, in line with the second and third steps of the pattern, the initial draft of the mental health program for the oocyte donors is designed and validated using the panel of experts and the classic Delphi technique. In the third phase, in line with fourth to sixth steps of the Talbot and Verrinder programing pattern, the program will be implemented and whose effectiveness will be evaluated.

### Phase I: qualitative study (evaluation of the needs and strategies of the program)

Using in-depth interviews, the qualitative study will be conducted to explain the needs and strategies of the program. The setting of this phase of the research is the centers for providing ART services in Isfahan. All of the interviews will be conducted in a private place selected based on the preference of the participants. The providers of ART services will also interviewed in their workplaces. After analyzing the qualitative data by the content analysis method, the proposed needs and strategies of the program will be extracted.

#### Participants

The participants of the qualitative phase of this research consist of the oocyte donors, the providers of ART services (midwives, gynecologist, psychiatrist, psychologist, and lawyer) and the family members of the oocyte donors who are willing to take part in the study. The participants will be selected using purposeful sampling method and considering the diversity of the age, education, number of their oocyte donation, and the motivation for entering the process of donation. After evaluating their inclusion criteria and obtaining their informed consent, the participants will be interviewed. The interviews will be continued until the repetition of the data and the feeling of reaching data saturation.

#### Qualitative data collection method

In the qualitative phase of the research, the data will be collected using in-depth interviews, taking notes in the field and reading the notebooks of the women donors. To observe ethical considerations, the participants are explained about the aims of the study and their informed consent will be obtained for recoding their voice. The setting and the length of the interviews will be specified based on the preferences of the participants. Data analysis will be performed using the conventional content analysis [17]. To assure the trustworthiness of the findings of the research, the four criteria of credibility, dependability, transferability and confirmability will be considered [18].

#### Inclusion and exclusion criteria

In the qualitative study, Iranian oocyte donors that a maximum of 6 months have passed from their entrance to the process of donation will be included in the study. Other inclusion criteria include no history of hospitalization or the use of medicine because of mental disorders, absence of stress, anxiety and severe depression in them, no history of ovarian hyper-stimulation syndrome in previous donations, and no legal problem related to donation. The inclusion criteria for the providers of donation services will be at least 2 years of work experience.

### Phase II: design and validation of the program

In this phase of the study, the priorities of the program will be developed based on the results of the first phase and the extracted strategies. Using review of the literature, the objectives and the operational program for achieving each objective are developed, and the validity of the program will be evaluated through using Delphi method in a study.

#### Holding a panel of experts

In this stage, in order to design the final version of the program and obtain the experts’ consensus of opinion, the initial draft of the program is designed and, then the classic Delphi technique is used for validating the program and determining its priorities. The members of the panel will consist of reproductive health specialists, planning experts, gynecologists, psychiatrists, sociologists, psychiatric nurses, psychologists, lawyers, and midwives. The electronic version of the program (and the hard copy if needed), together with open questions, will be sent to the members of the panel. These questions are to obtain the written opinion of the panel experts about the components of the program. After collecting the written opinion of the experts, content analysis will be performed and applying their opinions, the corrected version together with the assessment checklist is given to the experts. Then, collecting their opinions, the corrected version will be given to the experts. It is predicted that the program will be finalized during four rounds.

### Phase III: the implementation of the intervention program (quantitative study)

In this phase, the quantitative study as a multi-stage, two-group and field trial will be conducted on 72 oocyte donors.

#### Study sample

The setting of the research is the centers for providing ART services in Isfahan. Infertility and Shahid Beheshti fertility centers in Isfahan will consider as a research environment. The target population of the study consists of the oocyte donors at the time of the oocyte donation during the process of assisted reproductive treatment. Convenience sampling method will be used to select the subjects of the study.

#### Inclusion and exclusion criteria

Inclusion criteria at this phase of the study will include the absence of a known and under treatment physical illness, no oocyte donation during the past year, and no exposure to significant stressors during the past year based on Holmes and Rahe checklist (< 200 score); and if the ovulation induction is stopped because of the request of the recipient, the sample will be excluded from the study.

#### Data collection method

Before the implementation of the intervention group, the sampling process will be performed for the control group; then, the program is implemented and the sampling process will be done for the intervention group. The variables considered in this study will include depression, anxiety and stress levels in both groups during the three stages before the start of the oocyte donation, on the eighth day of the menstrual cycle (the time of the first follicle growth monitoring after ovulation induction) and after oocyte pick-up. To this end, the self-report DASS-21 questionnaire will be used. The DASS-21 questionnaire is a 21-item self-report tool in which each of the depression, anxiety and stress levels are assessed using seven items [19]. Each of the questions has a Likert scale of 0 to 3 with a range of 0 to 21 for each domain. The options are “never” (0), “little” (1), “sometimes” (2) and “always” (3). If the score obtained from the questions of the depression subscale ranges from 0 to 4, the subject will be in the normal range, 5–6 score means mild depression, 7–10 score suggests moderate depression, 11–13 severe depression, and 14 and over very severe depression. If the score of the anxiety subscale is between 0 and 3, the subject is in the normal range, 4–5 score suggests mild anxiety, 6–7 moderate anxiety, 8–9 means severe anxiety, while 10 and over shows very severe anxiety. Finally, if the score obtained from the questions of the stress subscale ranges between 0 and 7, the subject has a normal state, 8–9 shows a mild stress, 10–12 suggests moderate stress, 13–16 severe stress, and a score of 17 and over signifies very severe level of stress. The validity and reliability of DASS-21 have been confirmed for using in Iranian women [20].

#### Data analysis

The collected data will be analyzed using SPSS19 as well as descriptive and inferential statistical methods. Kolmogorov–Smirnov test will be used to evaluate the normality of the data. To compare quantitative baseline characteristics in the two groups of the control and intervention, independent t-test will be used, and for the comparison of the qualitative baseline characteristics, Mann-Whitney test will be used. Moreover, to compare nominal qualitative variables, chi-square will be used. Finally, repeated measures multivariate analysis of variance will be used to compare the mean of the mental health scores between the intervention and control group at the three measurement times.

## Discussion

The process of oocyte donation confronts the donors with social and ethical challenges that endanger their mental health. However, supportive programs can prevent the psychological injuries caused by such confrontations [21, 22]. Oocyte donation is a unique experience that the culture governing the provision of this service can create specific conditions. Therefore, mental health promotion programs for the target group and its needs assessment should be based on the experiences of the women involved in this process [21–23]. In designing this study, through analyzing the experiences of these women and the providers of donation services as well, there will be an attempt to identify the sufferings and feelings of the donors as well as the issues that may damage their mental health during the oocyte donation, and design the program based on them. Research findings also show that the programs which are based on need assessment are more successful [23, 24]. Attempts for the implementation of the interventions to improve the knowledge and awareness of the donors may also be associated with the reduction of psychological symptoms [25], but there is insufficient evidence in this regard.

However, the present program will be designed based on the socio-cultural and religious context of Iran and will provide robust information about the needs and strategies of the mental health promotion program for the women donors. As such, this program seems to be suitable for countries with similar characteristics such as the South Asian countries. Nonetheless, many of the obtained strategies may be applicable to other societies including Westerns countries. Thus, efforts will be made to develop strategies in such a way to be used in infertility centers, especially in countries with a socio-cultural structure like that of Iran. We hope that the designed program will have the capacity for being integrated into the instructions of ART service centers to take a step forward towards improving the mental health of the oocyte donors.

## Data Availability

Not applicable.

## Abbreviations

ART: Assisted Reproductive Techniques
DASS-21: Depression Anxiety Stress Scale-21
